# Stellettin B Induces G1 Arrest, Apoptosis and Autophagy in Human Non-small Cell Lung Cancer A549 Cells via Blocking PI3K/Akt/mTOR Pathway

**DOI:** 10.1038/srep27071

**Published:** 2016-05-31

**Authors:** Ran Wang, Qian Zhang, Xin Peng, Chang Zhou, Yuxu Zhong, Xi Chen, Yuling Qiu, Meihua Jin, Min Gong, Dexin Kong

**Affiliations:** 1 Tianjin Key Laboratory on Technologies Enabling Development of Clinical Therapeutics and Diagnostics, School of Pharmacy, Tianjin Medical University, Tianjin, 300070, China; 2 Research Center of Basic Medical Sciences, Tianjin Medical University, Tianjin, 300070, China; 3 State Key Laboratory of Toxicology and Medical Countermeasures, Beijing Institute of Pharmacology and Toxicology, Beijing, 100850, China

## Abstract

Until now, there is not yet antitumor drug with dramatically improved efficacy on non-small cell lung cancer (NSCLC). Marine organisms are rich source of novel compounds with various activities. We isolated stellettin B (Stel B) from marine sponge *Jaspis stellifera*, and demonstrated that it induced G1 arrest, apoptosis and autophagy at low concentrations in human NSCLC A549 cells. G1 arrest by Stel B might be attributed to the reduction of cyclin D1 and enhancement of p27 expression. The apoptosis induction might be related to the cleavage of PARP and increase of ROS generation. Moreover, we demonstrated that Stel B induced autophagy in A549 cells by use of various assays including monodansylcadaverine (MDC) staining, transmission electron microscopy (TEM), tandem mRFP-GFP-LC3 fluorescence microscopy, and western blot detection of the autophagy markers of LC3B, p62 and Atg5. Meanwhile, Stel B inhibited the expression of PI3K-p110, and the phosphorylation of PDK1, Akt, mTOR, p70S6K as well as GSK-3β, suggesting the correlation of blocking PI3K/Akt/mTOR pathway with the above antitumor activities. Together, our findings indicate the antitumor potential of Stel B for NSCLC by targeting PI3K/Akt/mTOR pathway.

Lung cancer is recognized as the most prevalent type of cancer and the leading cause of cancer death in the world^1^. Approximately 1.8 million new cases of lung cancer occurred, and about 1.59 million lung cancer patients died in 2012^2^. Non-small cell lung cancer (NSCLC) is the major type of lung cancer, accounting for more than 85% of cases^3^. Traditional chemotherapeutic drugs such as cisplatin cause serious side effects in patients, which are often direct causes of cancer death^4^. While molecular-targeted therapeutic drugs like Gefitinib (Iressa) was reported to increase 1-year survival rate to 29.9% of advanced NSCLC patients, such extent of efficacy is limited^5^. Therefore, novel drugs for NSCLC therapy are still expected.

Natural products have been the most prolific source of lead compounds for drug development^6^. Especially, antitumor agents from marine organisms have been a hot spot in pharmaceutical research^7^. The most important contribution for cancer therapy from the sea is cytarabine, which is currently used to treat leukemia and lymphoma^8^. Eribulin mesylate, an analogue of halichondrin B isolated from marine sponge, was approved for the treatment of metastatic breast cancer^9^. Over the past few years, about 3000 new compounds from various marine sources have been reported and some of them have entered clinical trials for cancer therapy^8^.

Marine sponges have served as one of the most productive sources of anticancer substances in marine environment^10^. As a part of our discovery of new anticancer agents from natural resources, we have been trying to search molecular-targeted antitumor lead compounds from marine sponges^11^,^12^,^13^,^14^. We isolated smenospongine from marine sponge *Dactylospongia elegans*, and reported the antiproliferative activities on leukemia cells and antiangiogenic effect on human umbilical vein endothelial cells (HUVEC)^13^. Aaptamine, a benzonaphthyridine alkaloid extracted from marine sponge *Aaptos suberitoids* by us, induced G2/M arrest in chronic myeloid leukemia K562 cells and activated p21 promoter in human osteosarcoma MG63 cells^12^,^14^. Recently, we isolated stellettin B (Stel B), an isomalabaricane triterpene from marine sponge *Jaspis stellifera*^15^. Interestingly, Stel B exhibited potent antiproliferative activities on various tumor cells including SF295 (IC50 = 0.01 μM), while showing very weak inhibition against four normal cell lines (IC50 > 10 μM)^15^.

In this paper we report the antitumor effects of Stel B on human NSCLC A549 cells, including the cell cycle G1 arrest, apoptosis and autophagy induction.

## Results

### Antiproliferative effect of Stel B on human NSCLC A549 cells

**Figure 1:**
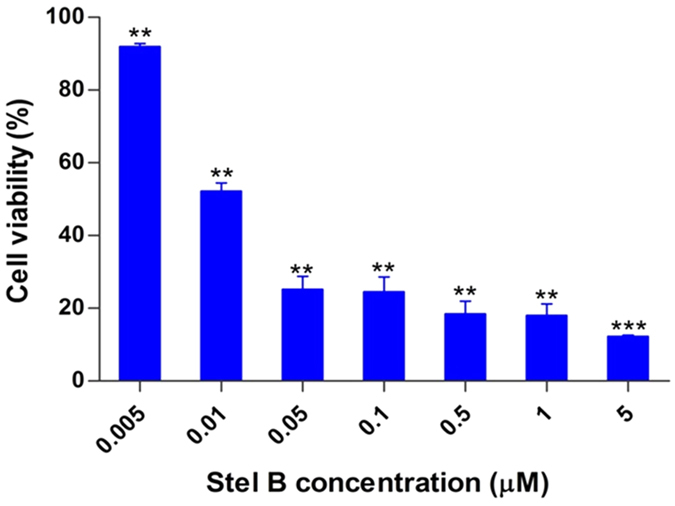
Stel B inhibited proliferation of A549 cells. Cell viability was determined by WST-8 assay after Stel B treatment at various concentrations (0, 0.005, 0.01, 0.05, 0.1, 0.5, 1 and 5 μM) for 48 h. The results are mean ± SD, representative of three independent experiments (n = 3). **p < 0.01, ***p < 0.001, compared with control.

The antiproliferative activity of Stel B on A549 cells was investigated by WST-8 assay, a sensitive colorimetric assay to determine cell viability by measuring dehydrogenase in living cells. After treatment by Stel B for 48 h, proliferation of A549 cells was inhibited in a dose-dependent manner (Fig. 1), with the IC50 value as 0.022 μM, suggesting the high inhibitory potency of Stel B. In addition, Stel B inhibited A549 proliferation time-dependently, with treatment for 72 h or longer at 5 μM leading to 100% inhibition (supplementary Fig. S1). And we preliminarily investigated whether A549 cells can develop resistance to Stel B. As a result, no obvious resistance occurred after ten days of exposure to 0.002 μM Stel B (supplementary Fig. S2).

### Stel B induced cell cycle arrest at G1 phase in A549 cells

**Figure 2:**
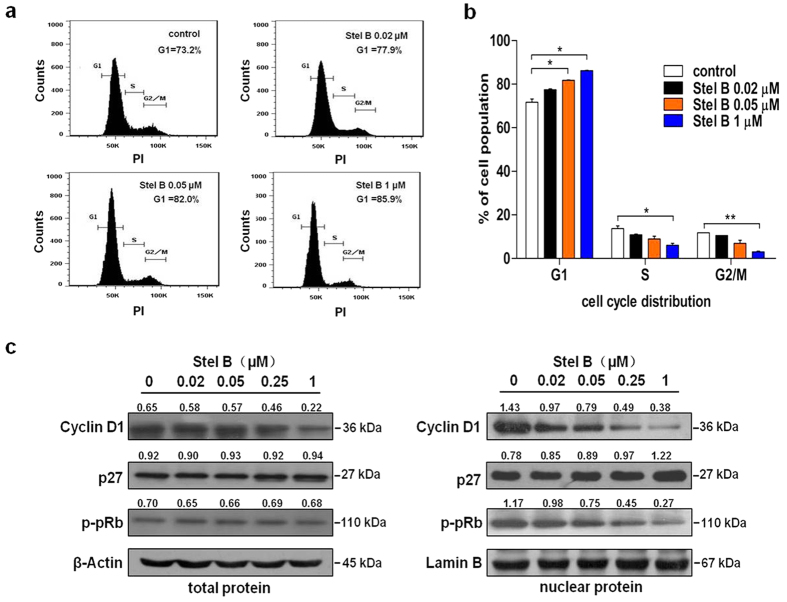
Stel B induced cell cycle arrest at G1 phase in A549 Cells. **(a)** Cell cycle distribution analysis by flow cytometer. A549 cells were incubated with indicated concentrations of Stel B for 24 h. The cells were collected, stained with PI and analyzed by flow cytometer. **(b)** The percentage of cell population at G1, S, and G2/M phases. Data are mean ± SD, representative of three independent experiments (n = 3). *p < 0.05, **p < 0.01, compared with the respective controls. **(c)** Effect of Stel B treatment on cell cycle-related proteins. A549 cells were treated with Stel B (0, 0.02, 0.05, 0.25, 1 μM) for 24 h. The levels of cyclin D1, p27, and p-pRb in total protein (left panel) as well as in nuclear protein (right panel) were determined by western blot.

Cell cycle is a repeating series of events that take place in a cell leading to its division and DNA replication to produce two daughter cells. Disturbance of cancer cell cycle would inhibit cell growth and activate apoptosis process. To examine whether Stel B affects cell cycle in A549 cells, we examined the cell cycle distribution after treatment with Stel B for 24 h. As shown in Fig. 2a, a dose-dependent accumulation of cells in G1 phase was observed after Stel B treatment. In contrast, the percentage of cells in S and G2/M phases reduced as Stel B concentration increased (Fig. 2b).

### Effect of Stel B on the expression of cell cycle-related proteins

Cell cycle is known to be regulated positively by cyclin-CDK (cyclin dependent kinase) complexes, but negatively by CDK inhibitors such as p27. To explore the potential molecular mechanism by which Stel B caused G1 arrest, we analyzed the expression levels in total and nuclear protein of the molecules which are known to be involved in G1/S checkpoint. Figure 2c and supplementary Fig. S4 show that Stel B significantly reduced the expression of cyclin D1 and phosphorylation of pRB, and increased the expression of CDK inhibitor p27, in nucleus of A549 cells. The level of cyclin D1 in the whole cell was downregulated as well. These results suggest that the G1 arrest induced by Stel B might be attributed to the effect on cyclin D1, p27 as well as pRb.

### Stel B induced apoptosis in A549 cells

**Figure 3:**
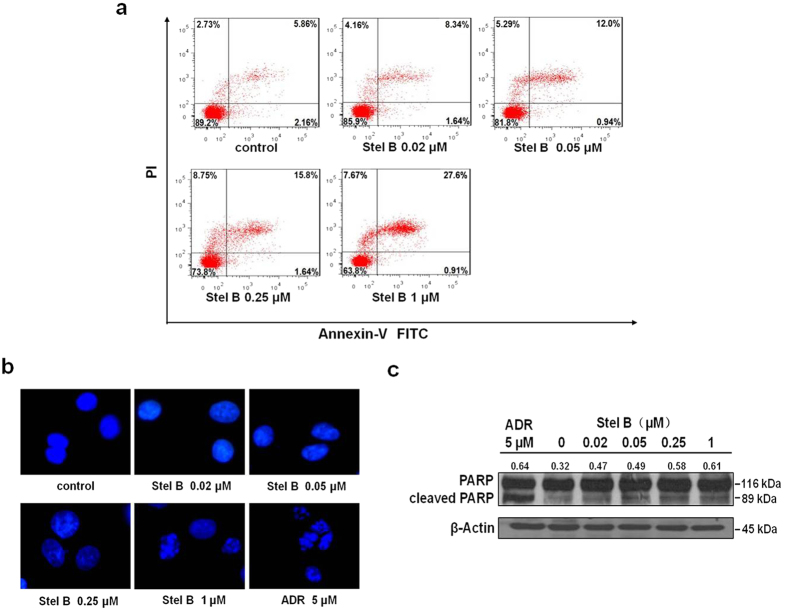
Stel B induced apoptosis in A549 cells. (**a)** Flow cytometric assay with Annexin V-FITC/PI double staining. A549 cells were treated with 0, 0.02, 0.05, 0.25 and 1 μM of Stel B for 24 h, stained with Annexin V-FITC and PI, then measured by flow cytometer. The population at upper-right quadrate (Annexin V^+^/PI^+^) increased to 8.34%, 12.0%, 15.8% and 27.6%, respectively, after treatment with 0.02, 0.05, 0.25, 1 μM of Stel B, compared to that for control cells (5.86%). **(b)** Fluorescence microscopy with DAPI staining. A549 cells were cultured together with Stel B (0, 0.02, 0.05, 0.25 and 1 μM) or ADR (5 μM) for 24 h. Then, the cells were stained with DAPI and observed under a fluorescence microscope. Cytoplasmic shrinkage and nuclear fragmentation occurred after treatment with 1 μM Stel B or 5 μM ADR. **(c)** Western blot analysis of cleaved PARP. A549 cells were treated with Stel B (0, 0.02, 0.05, 0.25, 1 μM) for 24 h. The levels of PARP and cleaved PARP were determined by western blot. The amount of cleaved PARP was increased in Stel B treated cells. ADR: Adriamycin.

To investigate whether Stel B induced apoptosis in A549 cells, we used Annexin V-FITC/PI double staining to measure the population of apoptotic cells. The population at upper-right quadrate (Annexin V^+^/PI^+^) increased to 8.34%, 12.0%, 15.8% and 27.6%, respectively, after treatment with 0.02, 0.05, 0.25, 1 μM of Stel B, compared to that for control cells (5.86%), suggesting that treatment with Stel B induced late-stage apoptosis in A549 cells (Fig. 3a).

To demonstrate the apoptotic induction, microscopy with DAPI staining was carried out. As shown in Fig. 3b, cytoplasmic shrinkage and nuclear fragmentation occurred after treatment with 1 μM of Stel B or 5 μM of Adriamycin (ADR), supporting that apoptosis was indeed induced.

As an important signaling protein involved in DNA repair and apoptosis, PARP is cleaved by upstream caspases when apoptosis occurs. Therefore, cleavage of PARP is widely used as a marker of apoptosis. We examined the effect of Stel B on PARP by western blot. As a result, the amount of cleaved PARP was increased in Stel B treated cells (Fig. 3c and supplementary Fig. S4), further confirming that Stel B induced apoptosis in A549 cells.

### Stel B increased reactive oxygen species (ROS) production

**Figure 4:**
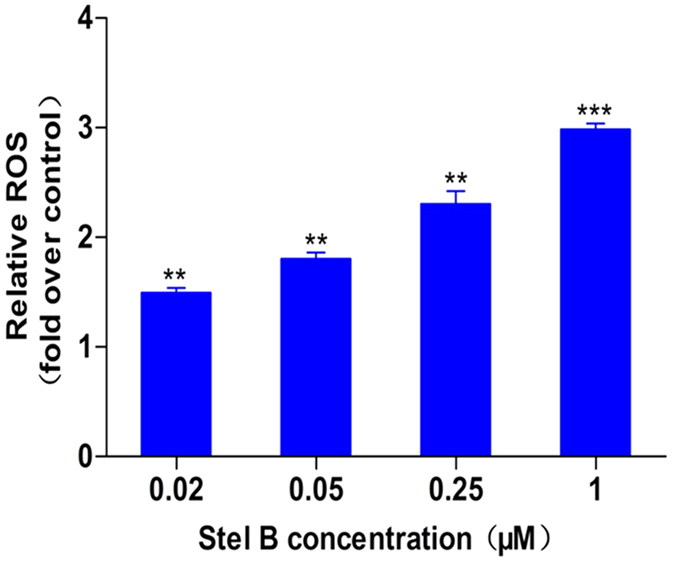
Stel B promoted ROS generation in A549 cells. A549 cells were treated with different concentrations of Stel B for 24 h, followed by DCFH-DA (10 μM) staining for 30 min. The ROS levels were determined by flow cytometer, and expressed as fold over that in untreated cells. Data are mean ± SD, representative of three independent experiments (n = 3). **p < 0.01, ***p < 0.001, compared with control.

By changing the internal environment of cells, ROS accumulation is known to play an important role in apoptosis^16^. We used DCFH-DA as a molecular probe to detect intracellular ROS production. As indicated in Fig. 4, the ROS level increased in a concentration-dependent manner following Stel B treatment, suggesting that Stel B promoted ROS generation in A549 cells.

### Stel B induced autophagy in A549 cells

**Figure 5:**
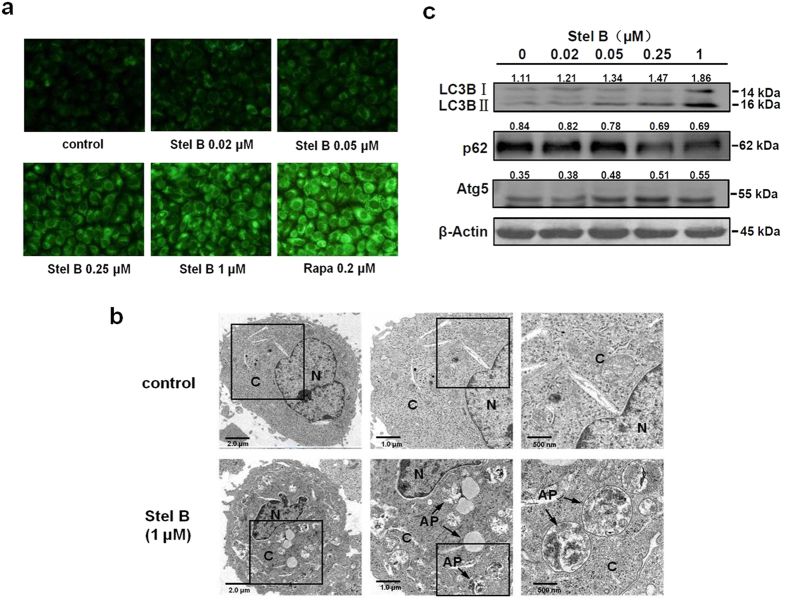
Stel B induced autophagy in A549 cells. **(a)** Fluorescence microscopy with MDC staining. A549 cells were treated with Stel B (0, 0.02, 0.05, 0.25, 1 μM) or 0.2 μM rapamycin (Rapa) for 24 h and then stained with MDC. The acidic vesicles were observed under a fluorescence microscope. MDC-stained vacuoles increased in Stel B or Rapa-treated cells, compared with those without treatment. **(b)** Transmission electron microscopy (TEM). A549 cells were cultured with or without 1 μM Stel B for 24 h and then subjected to TEM analysis. Autophagosomes (AP) are indicated in Stel B-treated A549 cells. Bars = 2.0 μm (left panel), 1.0 μm (middle panel) and 500 nm (right panel), respectively. N: nucleus; C: cytoplasm; AP: autophagosomes. **(c)** Western blot analysis of the levels of LC3B, p62 and Atg5 proteins. A549 cells were treated with Stel B (0, 0.02, 0.05, 0.25, 1 μM) for 24 h, and the expression levels of LC3B, p62, Atg5 and β-actin were determined by western blot.

Autophagy is a lysosomal degradation process for cytoplasmic constituents during the stress condition. To examine whether Stel B induced autophagy in A549 cells, several autophagic assays were performed. First, we used monodansylcadaverine (MDC) staining to detect autophagic vacuoles. As shown in Fig. 5a, Stel B induced the accumulation of MDC-labeled vacuoles in the cytoplasm in a dose-dependent manner. Rapamycin (Rapa), a well-known autophagy inducer, also obviously increased MDC-staining puncta production.

To elucidate cytological changes in autophagic cells induced by Stel B, we examined the intracellular morphologic change of A549 cells by use of transmission electron microscopy (TEM). As shown in the electron micrographs, a 450% increase of autophagic vacuoles which contain subcellular materials, were observed in Stel B-treated cells, compared with those in control cells (Fig. 5b). The size of autophagic vacuoles containing remnants of organelles was approximately 1 μm in diameter.

Moreover, we determined the effect of Stel B on the expression of autophagy marker proteins including LC3B, p62 and Atg5 by western blot. Amount of LC3B II/I was reported to be proportional to the number of the autophagic vacuoles^17^. Atg5 forms a conjugate with Atg12 and therefore plays a key role in autophagosome formation. P62 is a polyubiquitin-binding protein which contains a LC3-interacting motif and an ubiquitin-binding domain. By linking ubiquitinated substrate with autophagic machinery, p62 is incorporated in completed autophagosomes and degraded in autolysosomes, together with its bound proteins^18^. As shown in Fig. 5c and supplementary Fig. S4, the expression of LC3B II/I and Atg5 increased while that of p62 decreased after treatment with Stel B for 24 h, further confirming Stel B induced autophagy in A549 cells.

### Stel B promoted autophagic flux in A549 cells

**Figure 6:**
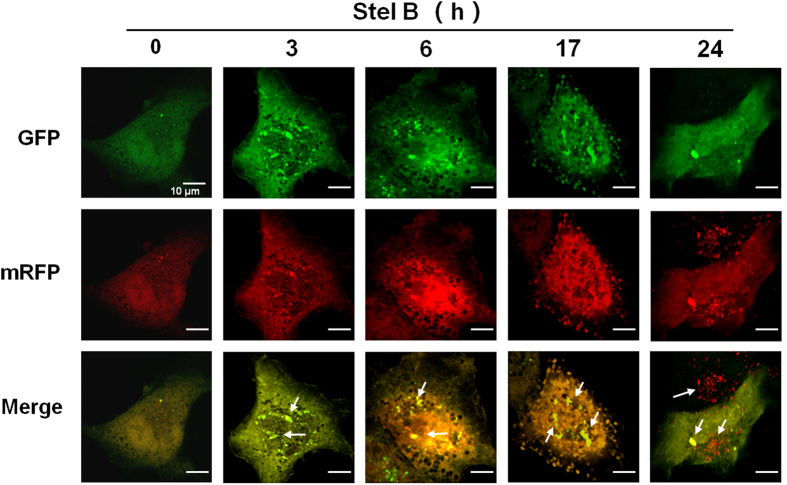
Stel B promoted autophagic flux in A549 cells. A549 cells were transfected with tandem fluorescent mRFP-GFP-tagged LC3 plasimd (ptfLC3) for 24 h. Then the transfected cells were treated with 1 μM of Stel B for 0, 3, 6, 17, 24 h, followed by confocal fluorescence microscopy. Arrows indicate autophagosomes. Bar = 10 μm. Data represent three independent experiments.

To elucidate the effect of Stel B on the autophagic process including autophagosome formation, fusion with and degradation in lysosome, we transfected A549 cells with a plasmid stably expressing mRFP (monomeric red fluorescent protein) - GFP (green fluorescent protein) - tagged LC3 (ptfLC3)^19^. At the early stage of autophagy, the ptfLC3 localizes to autophagosomes, developing both GFP (green) and mRFP (red) signals. The GFP protein is acid sensitive and quenched quickly following fusion of autophagosomes with lysosomes, whereas mRFP is relatively stable in the acidic environment of the autolysosome^20^. Before treatment with Stel B, only weak signals of GFP and mRFP protein which represent diffuse LC3 protein were found in the cytoplasm. After treatment with Stel B for 3 h, yellow puncta were observed in the perinuclear region, suggesting the formation of early autophagosomes. Sustained treatment until 17 h resulted in an increased number of autophagic vacuoles, suggesting that autophagosomes gradually developed maturing over time. At the time point of 24 h, the merge picture shows the accumulation of mRFP puncta and the decrease of GFP signal in Stel B treated A549 cells, indicating an increased autophagic flux (Fig. 6).

### Stel B inhibited PI3K/Akt/mTOR pathway via reducing p110 expression in A549 cells

**Figure 7:**
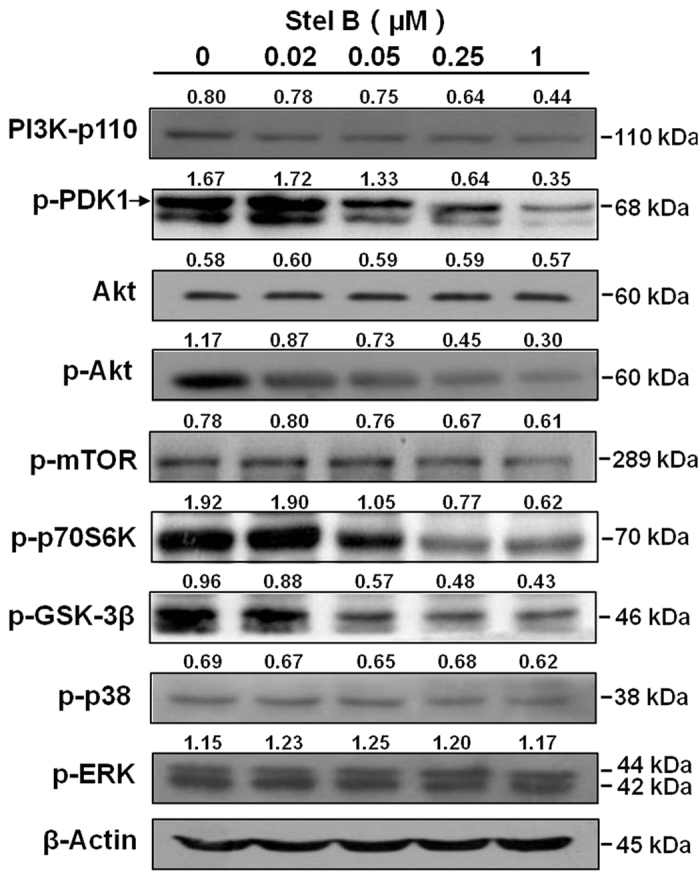
Stel B inhibited PI3K-p110 expression and the phosphorylation of the downstream effectors of PI3K/Akt/mTOR pathway. A549 cells were incubated with Stel B (0, 0.02, 0.05, 0.25 and 1 μM) for 24 h. The cells were harvested, and the cell lysates were prepared to be available for western blot analysis for PI3K-p110α, p-PDK1, p-Akt, Akt, p-mTOR, p-p70S6K, p-GSK-3β, p-p38 and p-ERK levels. PI3K-p110α expression was downregulated, and phosphorylation of PDK-1, Akt, mTOR, p70S6K, as well as GSK-3β was inhibited dose-dependently after Stel B treatment, while that of p38 and p-ERK was not affected obviously.

PI3K/Akt/mTOR pathway plays important roles in regulating cell cycle, cell apoptosis and autophagy. To investigate whether the effects of Stel B on A549 cells is related to this pathway, we examined the activity of Stel B on the representative signal proteins in the pathway. As shown in Fig. 7 and supplementary Fig. S4, phosphorylation of Akt, mTOR, p70S6K, as well as GSK-3β, was inhibited dose-dependently after Stel B treatment. Furthermore, phosphorylation of PDK1, which is the upstream activator of Akt was also blocked, implying the direct target of Stel B might be upstream molecules of PDK1. More interestingly, Stel B inhibited the expression of p110, the catalytic subunit of PI3K, in a dose-dependent manner, suggesting that Stel B blocked PI3K/Akt/mTOR pathway via reducing PI3K-p110 expression. In addition, Stel B was demonstrated to preferentially inhibit PI3K/Akt pathway, since the phosphorylation of p38 and ERK, which are downstream effectors of Ras/MAPK pathway, was not affected (Fig. 7 and supplementary Fig. S4).

## Discussion

Marine organisms contain a great number of novel bioactive compounds with potential to be developed as new drugs. To date, fourteen stellettin analogs were isolated from various marine sponges and some of them showed cytotoxic activities^21^,^22^,^23^,^24^. We previously reported Stel B induced apoptosis in neuroblastoma SF295 cells^15^. In this study, we reported for the first time the multifaceted *in vitro* antitumor activities on A549 cells. Stel B exhibited potent antiproliferative activity on A549 cells with an IC50 as 0.022 μM. G1 arrest, apoptosis as well as autophagy were induced by Stel B treatment.

Cell proliferation needs cell cycle progression, which is known to be controlled by cyclin-CDK complex and CDK inhibitor proteins. In G1/S checkpoint, cyclin D1 forms a complex with CDK4, and therefore inhibits pRb via phosphorylation, resulting in the release of E2F to promote progression through G1 phase^25^. On the other hand, the activity of CDK4-cyclin D1 complex is negatively controlled by CDK inhibitor proteins including p27^26^. Treatment by Stel B caused reduction in expression of cyclin D1 and phosphorylation of pRb, and enhancement in p27 expression. Therefore, Stel B-induced G1 arrest might be attributed to downregulation of CDK4-cyclin D1 complex and upregulation of p27.

Induction of apoptosis highly affects cell proliferation. Flow cytometry with Annexin V/PI staining suggested that Stel B induced apoptosis in A549 cells, which was supported by the result of DAPI staining assay and increased amount of cleaved PARP. In addition, Stel B significantly promoted ROS generation in A549 cells. It is known that ROS over-production can induce oxidative stress, resulting in apoptosis^27^. Therefore, promotion of ROS generation by Stel B might lead to apoptosis, which could contribute to the antitumor effect of Stel B.

Autophagy is an evolutionarily self-digesting process in which cytoplasmic material is sequestered within cytosolic double-membraned vesicles-autophagosomes, and ended up in the lysosome^28^. In order to investigate the effect of Stel B on autophagy, we utilized various assay methods. MDC staining and TEM showed the formation of autophagosomes. Western blot analysis indicated that the levels of autophagy marker LC3B II/I and Atg5 were increased and the level of p62 was decreased. We also used Tandem mRFP-GFP-LC3 fluorescence assay to confirm the autophagic flux in Stel B-treated cells. As another type of cell death besides of apoptosis, autophagy was frequently reported to be induced by many antitumor agents including taxanes and molecular-targeted agents^29^,^30^. On the other hand, autophagy was reported to enhance production of ATP, which subsequently binds purinergic receptor P2RX7 in dendritic cells (DC), stimulates the recruitment of DC into the tumor bed, and finally leads to the immunogenic cell death (ICD) of tumor cells^31^,^32^, suggesting the autophagy induced by Stel B might contribute to the antitumor efficacy.

Finally, we investigated the mechanism which might be involved in the above effects of Stel B. We previously reported that Stel B inhibited phosphorylation of Akt in SF295 cells^15^. Therefore, the effect of Stel B on Akt pathway was examined in A549 cells. As expected, phosphorylation of Akt and the downstream effectors including mTOR, p70S6K and GSK-3β, was inhibited in a dose-dependent manner. Akt is known to increase cyclin D1 through inactivation of GSK-3β and reduce p27 by inhibition of Forkhead family transcription factors and the tumor suppressor tuberin (TSC2)^33^. Therefore, induction of G1 arrest by Stel B might be attributed to the influence on GSK-3β as well as the upstream Akt. It is well known that Akt pathway plays a key role in cell survival, therefore, the apoptosis induced by Stel B might be attributed to the inhibition of Akt phosphorylation. As a downstream effector of Akt, mTOR is known to negatively control autophagy^34^, and mTOR inhibitor rapamycin is well reported as an autophagy inducer^17^. Stel B inhibited phosphorylation of mTOR and p70S6K at a similar concentration to that for autophagy induction in A549 cells, suggesting the autophagy-inducing effect might be attributed to the inhibition of Akt/mTOR pathway.

In order to investigate the target of Stel B in A549 cells, we determined the activity of Stel B on the upstream activators of Akt. As an upstream of Akt and downstream of phosphatidylinositol 3,4,5-trisphosphate (PIP3), PDK1 is phosphorylated by PIP3 and subsequently phosphorylates Akt at Ser308. Phosphatidylinositol 3-kinases (PI3Ks), which contain a catalytic subunit p110 and a regulatory subunit, phosphorylate the 3-hydroxyl group of phosphatidylinositol 4,5-bisphosphate (PIP2) to generate PIP3. Our results showed that Stel B treatment inhibited the phosphorylation of PDK1, and the expression of p110 (Fig. 7). Therefore, the G1 arrest, apoptosis and autophagy inducing effects of Stel B might be attributed to p110 reduction, which leads to inhibition of the downstream effectors like PDK1, Akt, mTOR, as well as GSK-3β.

In conclusion, we isolated Stel B from marine sponge *Jaspis stellifera*. Stel B treatment induced G1 arrest, apoptosis and autophagy in A549 cells, in which reduction of PI3K-p110 expression and consequent inhibition of PI3K/Akt/mTOR pathway might be closely involved. Since Stel B showed potent activities on human NSCLC A549 cells at low nM doses, and our previous report indicated its very weak cytotoxicity on normal cell lines^15^, it might become a promising drug candidate for NSCLC cancer therapy in the future. However, we realize that it is necessary to exhibit favorable *in vivo* antitumor activity for stellettin B to become a drug candidate, which remains unclear and will be investigated in our next work.

## Materials and Methods

### Reagents

WST-8 assay kit was purchased from Dojindo Laboratories (Kumamoto, Japan). FITC Annexin V Apoptosis Detection Kit, and antibodies against phospho-Rb (S780), p27, as well as cyclin D1 were obtained from BD Biosciences (San Jose, CA, USA). Antibodies against phospho-GSK-3β (Ser9), Akt, phospho-Akt (Ser473), phospho-PDK1 (Ser241), phospho-mTOR (Ser2448), phospho-p70S6K (Thr389), PI3K-p110α, PARP, LC3B, SQSTM1/p62, Atg5, phospho-p38 (Thr180/Thr182), phospho-ERK1/2 (Thr202/Thr204) and β-actin were purchased from Cell Signaling Technology (Danvers, MA, USA). Antibody against Lamin B, and 4′, 6-diamidino-2-phenylindole (DAPI) were obtained from Santa Cruz Biotechnology (Santa Cruz, CA, USA). Monodansylcadaverine (MDC), Propidium Iodide (PI), and 2′,7′-Dichlorofluorescein diacetate (DCFH-DA) were purchased from Sigma-Aldrich (St. Louis, MO, USA). ptfLC3 was a gift from Tamotsu Yoshimori (Addgene plasmid #21074, Cambridge, MA, USA). Lipofectamine 2000 was obtained from Life Technologies (Carlsbad, CA, USA).

### Cell culture

Human non-small cell lung cancer A549 cells were obtained from Cell Resource Center, Peking Union Medical College (Beijing, China). The cells were cultured in a humidified incubator with an atmosphere containing 5% CO_2_ at 37 °C, and maintained in RPMI1640 medium supplemented with 10% FBS and antibiotics (0.1 μg/ml of penicillin and 0.1 μg/ml of streptomycin).

### Isolation and structure identification of Stel B

Stel B was isolated from the marine sponge *Jaspis stellifera*, as described by us previously^15^. Chemical structure of the compound was identified by comparison of the mass and NMR data with those reported previously^35^. The purity of Stel B is 99%.

### Cell viability assay

WST assay was used to determine the inhibitory effect of Stel B on the proliferation of A549 cells, as described by us previously^36^. Briefly, cells were cultured in 96-well plates at 37 °C with 0.005, 0.01, 0.05, 0.1, 0.5, 1 and 5 μM of Stel B for 48 h. Then, 10 μl of WST-8 was added and further incubated for 4 h. The resulting absorbance at 450 nm was measured by using microplate reader iMark (Bio Rad, Hercules, CA, USA).

### Cell cycle distribution analysis

The effect of Stel B on cell cycle distribution was analyzed by flow cytometer with PI staining as we previously described^12^. Briefly, A549 cells were incubated in 6-well plates with 0, 0.02, 0.05 and 1 μM of Stel B for 24 h. The cells were collected, fixed in 75% ethanol, and resuspended in 50 μg/ml of PI solution. The treated cells were subjected to flow cytometer FACS Verse (Becton Dickinson, Germany) for cell cycle distribution analysis. Data were quantified by using Flow Jo Software (Tristar, CA, USA).

### Flow cytometric analysis of apoptosis with Annexin V/PI staining

Apoptosis analysis was carried out by detecting phosphatidylserine (PS) externalization using flow cytometer as we reported previously^15^, with a small modification. Briefly, A549 cells were cultured together with 0, 0.02, 0.05, 0.25 and 1 μM of Stel B for 24 h in 6-well plates. Next day, cells were collected, washed with ice-cold PBS, and resuspended in 50 μl of binding buffer containing Annexin V-FITC and PI. Then cells were incubated for 15 min in the dark. After dilution, the samples were available for analysis of apoptosis, using flow cytometer FACS Verse (Becton Dickinson, Germany). Data were quantified by using Flow Jo Software (Tristar, CA, USA).

### DAPI staining

DAPI staining assay was carried out to observe morphological characteristics of apoptotic cells. A549 cell suspension was plated on the coverslips in 6-well plates at a density of 5 × 10^5^ cells/well, followed by treatment with 0, 0.02, 0.05, 0.25 and 1 μM of Stel B for 24 h. ADR, an apoptosis inducer, was used as a positive control. Then, the cells were washed with PBS, fixed with 4% paraformaldehyde, permeabilized with 0.1% Triton X-100, and then stained with 1 μg/ml of DAPI solution for 10 min. The fluorescent images of treated samples were obtained using DMI3000B fluorescent microscope (Leica, Germany) with LAS V4.3 software.

### Measurement of intracellular ROS generation

ROS measurement was performed by using DCFH-DA, which was de-esterified intracellularly and became highly fluorescent 2′,7′-dichlorofluorescein upon oxidation^37^. A549 cells grown on 6-well plates were treated with 0, 0.02, 0.05, 0.25 and 1 μM of Stel B for 24 h. The cells were harvested, washed with PBS, and then incubated with 10 μM of DCFH-DA in the dark at 37 °C for 30 min. The fluorescent signal produced was analyzed by using flow cytometer FACS Verse (BD, Germany).

### MDC staining

MDC, a specific marker for autophagic vacuoles, was used to examine whether Stel B induced autophagy. A549 cells were grown on the coverslips in 6-well plate, treated with 0, 0.02, 0.05, 0.25 and 1 μM of Stel B for 24 h. Rapamycin was used as a positive control. The cells were washed with ice-cold PBS, and incubated with 50 μM of MDC at 37 °C for 30 min. The stained cells were washed, fixed with 4% paraformaldehyde, and analyzed by fluorescence microscope BX51 (Olympus, Japan) with MetaMorph software.

### Transmission electron microscopy (TEM)

As the most reliable approach for monitoring autophagy, TEM was utilized to confirm autophagy^17^. A549 cells were cultured in 6 cm dishes with 1 μM of Stel B for 24 h. Cells were collected and fixed. The ultrathin 50 nm sections were cut by use of an ultramicrotome, stained with 2% (w/v) uranyl acetate and lead citrate, then examined with electron microscope Hitachi 600 (Hitachi, Japan).

### Analysis of autophagic flux

To analyze autophagic flux, A549 cells were transfected with a tandem fluorescent mRFP-GFP-tagged LC3 plasmid^20^ using lipofectamine 2000 according to the manufacturer’s instructions. The transfected cells were then treated with 1 μM of Stel B for 0, 3, 6, 17 and 24 h. The expression of GFP and mRFP was visualized with Olympus FV1000 laser scanning confocal microscope (Olympus, Japan). Images were acquired by using FV10-ASW3.0 software. Autophagic flux was evaluated by the color change of GFP/mRFP at different time points.

### Western blot analysis

Western blot analysis was performed as we described previously^38^ with a small modification. A549 cells were treated with 0, 0.02, 0.05, 0.25 and 1 μM of Stel B for 24 h. To prepare the whole cell lysate, the cells were harvested, and lysed with RIPA buffer. For detection of nuclear proteins such as pRb, p27 and cyclin D1, nucleus lysate was prepared using NE-PER Nuclear and Cytoplasmic Extraction Kit (Thermo Scientific, Rockford, IL, USA). Equivalent amounts of protein were loaded and separated by sodium dodecyl sulfate-polyacrylamide gel electrophoresis (SDS-PAGE) and then transferred to Immobilon-PSQ PVDF (polyvinylidene fluoride) membrane. The membranes were blocked in 5% non-fat milk, incubated with primary antibodies, followed by exposure to the respective HRP (horseradish peroxidase) - conjugated secondary antibodies. Immunoreactive bands were visualized using ECL (enhanced chemiluminescence) reagents and digitalized by scanning. The level of each protein has been quantified using the software of image J (NIH, Bethesda, MD, USA), followed by densitometric analysis of the ratio of the density of each target protein to the β-actin (for total protein) or lamin B (for nuclear protein) band.

### Statistical analysis

Data are presented as mean ± standard deviation (SD), representative of at least three independent experiments. Student’s *t*-test was carried out with GraphPad Prism 5 software (GraphPad, San Diego, CA, USA). The p value < 0.05 was considered statistically significant.

## Additional Information

**How to cite this article**: Wang, R. *et al.* Stellettin B Induces G1 Arrest, Apoptosis and Autophagy in Human Non-small Cell Lung Cancer A549 Cells via Blocking PI3K/Akt/mTOR Pathway. *Sci. Rep.* **6**, 27071; doi: 10.1038/srep27071 (2016).

## Supplementary Material

Supplementary Information
