# Supplementary material for: Stellettin B Induces G1 Arrest, Apoptosis and Autophagy in Human Non-small Cell Lung Cancer A549 Cells via Blocking PI3K/Akt/mTOR Pathway
Source: Sci Rep. 2016 May 31;6:27071. doi: 10.1038/srep27071 (PMC4886687; doi:10.1038/srep27071)
Supplement: Supplementary Information [file srep27071-s1.pdf]

Supplementary information

Stellettin B Induces G1 Arrest, Apoptosis and Autophagy in Human Non-small Cell Lung Cancer A549 Cells via Blocking PI3K/Akt/mTOR Pathway

Ran Wang[1], Qian Zhang[1,2], Xin Peng[1,2], Chang Zhou[1,2], Yuxu Zhong[3], Xi Chen[1,2], Yuling Qiu[1], Meihua Jin[1], Min Gong[1], and Dexin Kong[1,2,*]

[*]Corresponding author: Dexin Kong

Supplementary Figure 1. (Fig. S1) Stel B time-dependently inhibited proliferation of A549 cells.

Supplementary Figure 2. (Fig. S2) Pretreatment of A549 cells with low concentration of Stel B did not lead to obvious resistance.

Supplementary Figure 3. (Fig. S3) Uncropped blots of all western blot results.

Supplementary Figure 4. (Fig. S4) Statistical analysis of all western blot results.

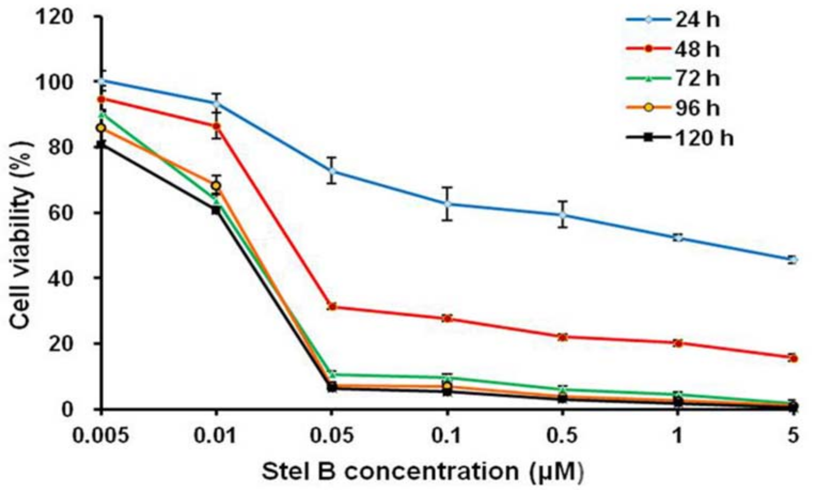

Fig. S1. Stel B time-dependently inhibited proliferation of A549 cells. Cell viability was determined by WST-8 assay after Stel B treatment at various concentrations (0, 0.005, 0.01, 0.05, 0.1, 0.5, 1 and 5 μM) for 24, 48, 72, 96 and 120 h, respectively. Treatment for 72 h or longer with 5 μM of Stel B led to 100% inhibition of A549 proliferation. Data are mean ± SD, representative of three independent experiments (n=3).

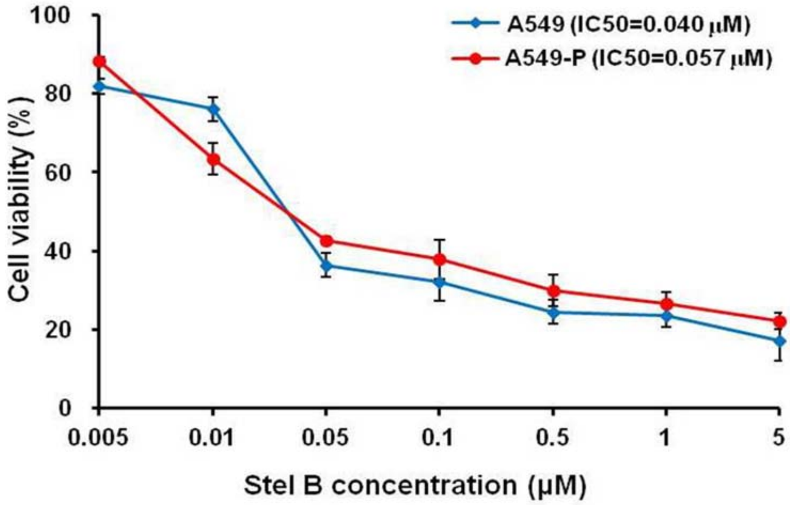

Fig. S2. Pretreatment of A549 cells with low concentration of Stel B did not lead to obvious resistance. To examine whether A549 cells can develop resistance to Stel B, A549 cells were exposed to 0.002 μM of Stel B (10% of IC50) for 10 days, followed by treatment with higher concentrations (0.005, 0.01, 0.05, 0.1, 0.5, 1 and 5 μM) of Stel B for 48 h. Then, WST-8 assay was performed to detect cell viability, using these Stel B-pretreated A549 cells (A549-P) and cells without pretreatment. As a result, no obvious decrease in sensitivity to Stel B was found in A549-P cells (IC50: 0.057), compared to those without pretreatment (IC50: 0.040), suggesting A549 cells might not develop resistance to Stel B in the present condition. Data are mean ± SD, representative of three independent experiments (n=3). A549-P: Stel B pretreated A549 cells.

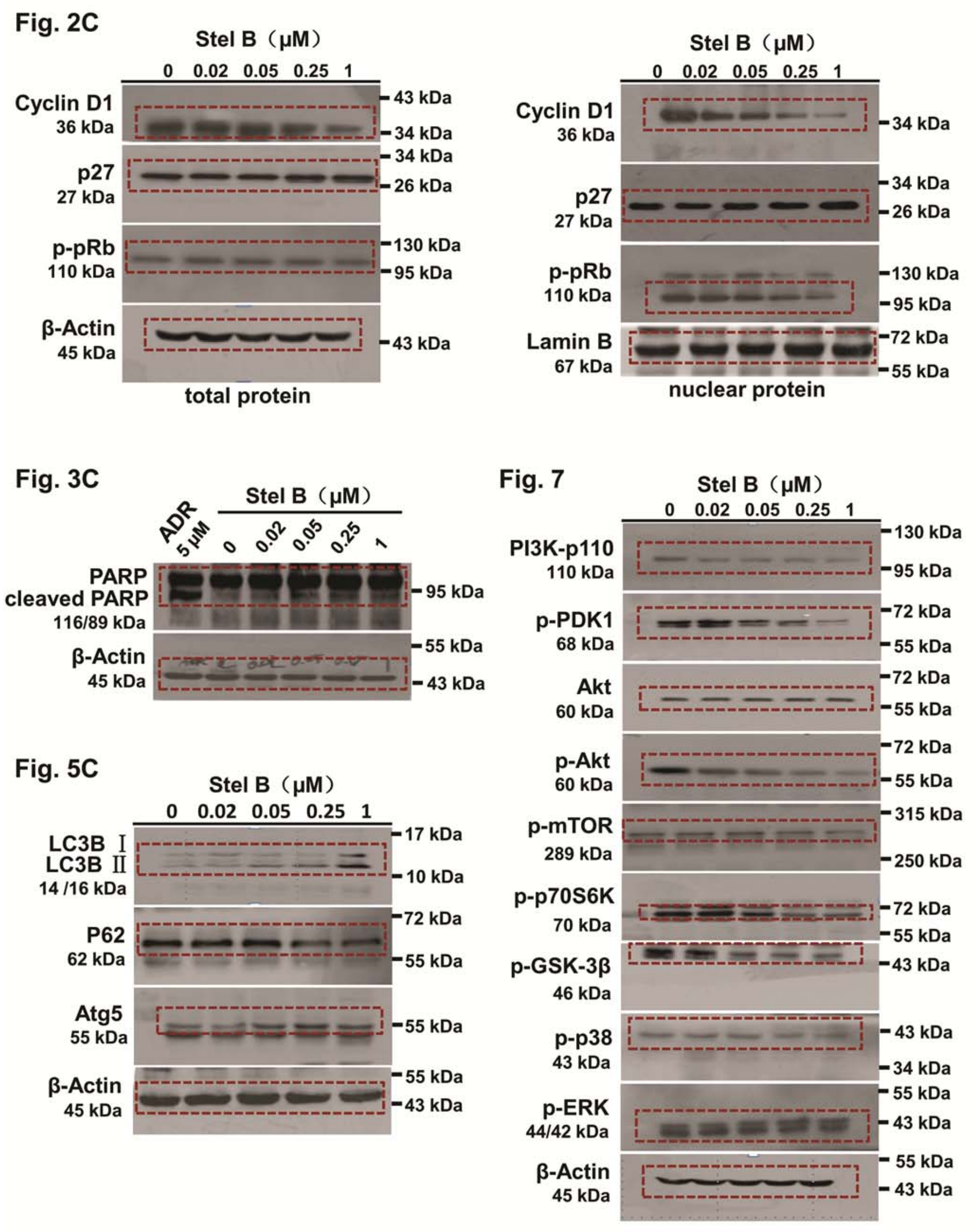

Fig. S3. Uncropped blots of all western blot results.

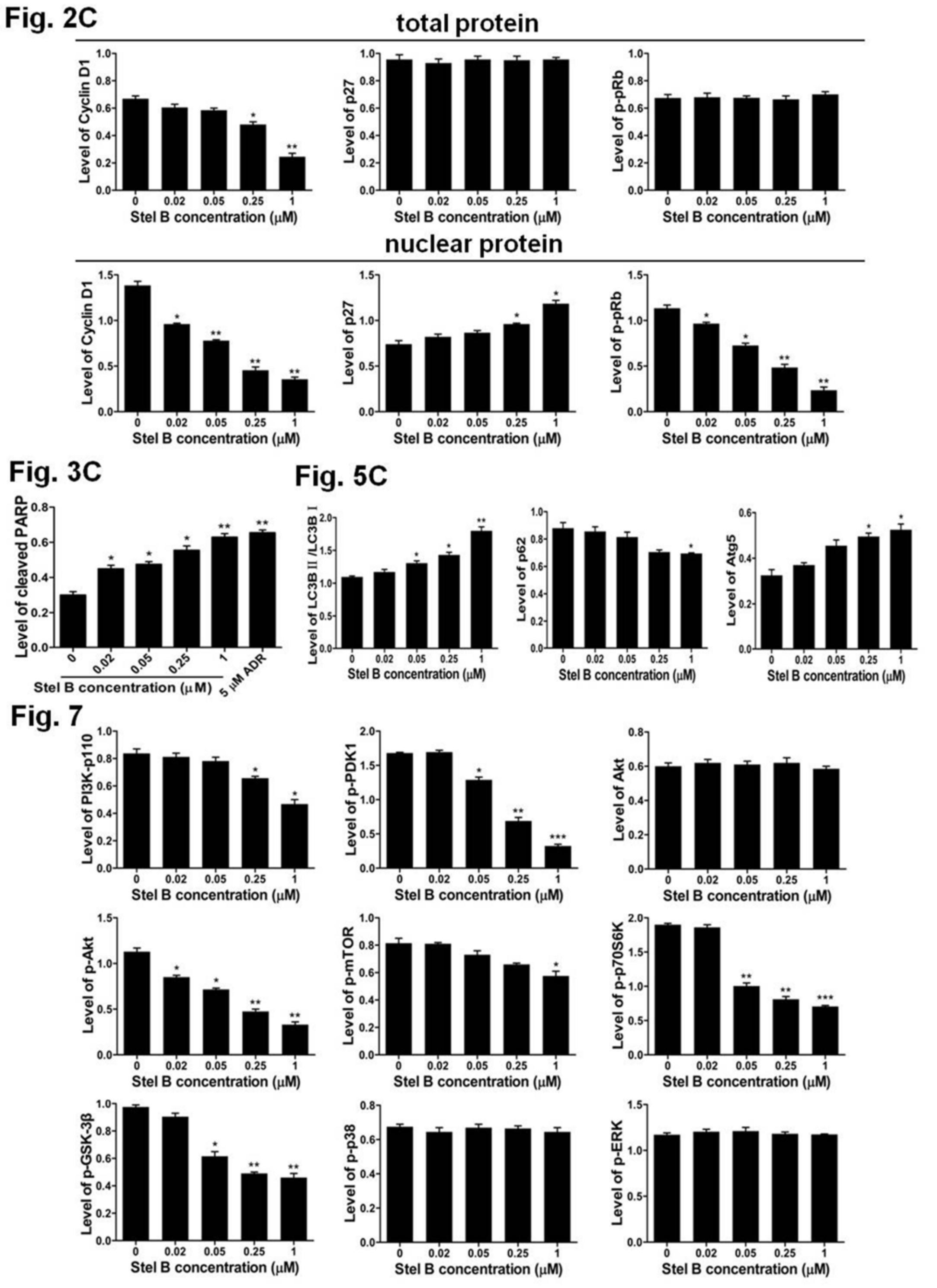

Fig. S4. Statistical analysis of all western blot results. Bar graphs show the relative levels of Cyclin D1, p27, p-pRb, cleaved PARP, LC3B Ⅱ/LC3B I, p62, Atg5, PI3K-p110, p-PDK1, Akt, p-Akt, p-mTOR, p-p70S6K, p-GSK-3β, p-p38 and p-ERK in A549 cells treated with 0, 0.02, 0.05, 0.25 and 1 μM of Stel B. Student's *t*-test was

carried out for statistical analysis by use of GraphPad Prism 5 software (GraphPad, San Diego, CA, USA). Data are mean ± SD, representative of three independent experiments. *: $p < 0.05$, **: $p < 0.01$, ***: $p < 0.001$, compared with untreated (0 μM of Stel B) A549 cells.
